# Preparation and Application of Cellulose-Based Materials with Selective Adsorption of Dyes

**DOI:** 10.3390/polym17121653

**Published:** 2025-06-14

**Authors:** Linlin Bai, Yuxing Chen, Huiting Ma, Xu Meng

**Affiliations:** 1 School of Textile Science and Engineering, Cai Yuanpei School of Art and Design, Shaoxing University, Shaoxing 312000, China; bailinlin0210@163.com (L.B.); 13858957925@163.com (H.M.); 2 School of Materials Science and Engineering, Changzhou University, Changzhou 213164, China; 18370141946@163.com

**Keywords:** cellulose, dye adsorption, wastewater purification, environment, degradation

## Abstract

A cellulose-based material with high adsorption capacity and surface area was developed by selecting appropriate copolymer monomers for structural design. This material was used for selective dye adsorption in wastewater treatment. The copolymer was characterized by scanning electron microscopy (SEM), thermogravimetric analysis (TGA), and Fourier-transform infrared spectroscopy (FTIR) to investigate its microstructure, structure, thermal stability, and thermal decomposition. We explored the factors affecting dye adsorption, including dye type, adsorption reaction time, initial dye concentration, copolymer dosage, temperature, and the acidity or alkalinity of the reaction environment. The results showed that as the adsorption reaction time increased, the amount of adsorbed Rhodamine B dye gradually increased, and the initial stage (0–20 min) increased rapidly. When the initial dye concentration was 15 mg/L, the adsorption capacity (*q_e_*) was at its maximum (3.67 mg/g). In addition, when the amount of copolymer used was 5 mg/10 mL, the adsorption capacity (*q_e_*) was the highest (12.37 mg/g). High-temperature conditions were favorable for adsorption, with the maximum adsorption capacity (*q_e_*) at 35 °C (13.48 mg/g). The prepared copolymer exhibited significant adsorption performance in acidic environments (pH = 3). The polymer adsorbed with dye was degraded by UV irradiation, avoiding secondary pollution caused by recycling.

## 1. Introduction

In recent years, due to the rapid development of industries such as textile finishing, plastics, papermaking, cosmetics, pharmaceuticals, and food processing, organic dye pollutants have attracted increasing attention [1,2,3,4]. The chemical industry consumes over 7 × 10^5^ tons of dyes annually [5], and it is reported that 2% of the dyes produced each year are discharged into water resources during industrial processing. Undoubtedly, dyes are the most direct source of pollution. The wastewater obtained from the textile industry not only has a large discharge volume but also usually contains deep and persistent colors, as well as high chemical oxygen demand (COD) and total dissolved solids (TDS).

Textile wastewater is often rich in complex chemicals and inorganic salts, and its pH, temperature, turbidity, salinity, and water quality frequency vary greatly [6]. However, there are various highly complex chemical substances in textile wastewater, including toxic and harmful substances such as degreasing solvents, alkaline cleaners, cyanides, oils, and metals [7]. Directly releasing textile wastewater into the environment is inevitably harmful, not only because it has adverse effects on the appearance, transparency, and dissolved oxygen content of freshwater bodies [8], but also because it contains many dyes and their degradation products, including organic compounds based on aromatic compounds, which have highly stable complex aromatic structures, high molecular weight, low biodegradability, and are stable to heat, light, and oxidants, making them difficult to degrade in natural environments [7].

Moreover, they have potential bioaccumulation hazards, including mutagenicity, carcinogenicity, reproductive and developmental toxicity, neurotoxicity, and chronic toxicity to organisms [9,10]. They can also irritate the skin, eyes, and respiratory tract, and they may cause various diseases in humans, especially respiratory system diseases such as hemolysis, hypertension, jaundice, organ damage, tissue necrosis, etc. [11]. Once textile wastewater accumulates for a long time, it may also have potential adverse effects on soil and plant systems. Therefore, it is necessary to remove harmful dye molecules from textile wastewater before discharging it.

At present, many physical, chemical, and biochemical treatment processes have been extensively studied for the treatment of dye pollutants, such as coagulation, flocculation, ion exchange [12], membrane separation [13], oxidation/ozonation [14], biodegradation [15], chlorination, photocatalytic degradation [16], and adsorption [17]. However, traditional processing techniques still have many vulnerabilities. For example, biological methods not only take a considerable amount of time but also cannot degrade complex dyes [18]. In addition, some commercial dyes are harmful to certain microorganisms [19]. Chemical coagulation can even lead to the formation of colloids in wastewater, causing pollution to water bodies, etc. [20]. However, common chemical reagents such as chlorine gas have a slow oxidation reaction rate and require reactive materials during the reaction, making them very dangerous for transportation and storage [21]. It cannot be ignored that, due to the presence of multiple materials in wastewater [22], the efficiency of these methods is not high. Relatively speaking, more advanced oxidation methods, such as ozone oxidation, photocatalysts, and photocatalytic Fenton processes, are both expensive and uneconomical [18].

Currently, adsorption technology is widely recognized as one of the most convenient and effective methods for pollutant removal, due to its low initial investment, simple design, easy operation, high efficiency, and high productivity. However, traditional adsorbents have disadvantages such as expensive regeneration, poor selectivity, and high loss rates. For example, the main problems with inorganic minerals and natural materials are related to their poor selectivity, high regeneration costs, and weak adsorption capacity [23]. Porous resins and activated carbon are commonly used as adsorbents, but these adsorbents are difficult to recover after use, which to some extent limits their widespread application. Due to the high costs and energy required for producing adsorbents, scientists have been motivated to actively develop new alternatives, namely, alternative adsorbents with high adsorption capacity, selectivity, and rapid regeneration rates. Therefore, adsorbents with unique advantages, such as abundant raw materials, simple preparation, high efficiency, low cost, environmental friendliness, and easy recycling, have attracted much attention [6].

Due to their high surface area, excellent mechanical stiffness, adjustable surface chemical properties, and easy regeneration under mild conditions, polymer adsorbents have become potential alternatives to activated carbon, especially those that can be easily regenerated through a simple and non-destructive washing process and used in cyclic adsorption processes [23]. Hydrogels obtained from biological resources, such as sodium alginate, starch, cellulosic materials [24,25], and deacetylated chitosan, are becoming potential alternative adsorbents for synthetic dyes of metal ions and water-based dyes. In recent years, cellulose-based materials have become adsorbents for removing organic pollutants and heavy metals from wastewater due to their renewable, sustainable, biodegradable, and anionic properties, and they have gradually been widely studied. The use of cellulose-based materials in aqueous solutions as bio-adsorbents that can capture dyes will increasingly attract people’s attention [26]. This work uses cellulose with a high adsorption capacity and high surface area as the skeleton and, through structural design, selects copolymer monomers to prepare materials with selective adsorption of dyes, which are used for dye wastewater treatment.

## 2. Materials and Methods

### 2.1. Materials

Sodium carboxymethyl cellulose (CMC), N, N′-methylenebisacrylamide (MBA), potassium 3-sulfonate propyl methacrylate (SPMA), 2-hydroxy-4′-(2-hydroxyethoxy)-2-methylphenylacetone, nano-titanium dioxide (TiO_2_), Rhodamine B, and soap were purchased from Shanghai Aladdin Reagent Co., Ltd., Shanghai, China.

### 2.2. The Preparation Principle and Process of Polymers

**Figure 1:**
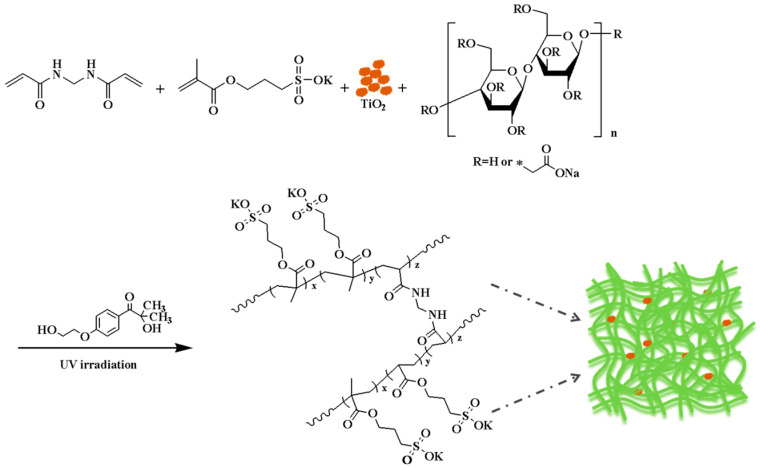
Reaction equation of copolymer P(CMC-SPMA-MBA).

The gel-like solid was obtained by the reaction of three monomers, namely, CMC, MBA, and SPMA, initiated by 2-hydroxy-4′-(2-hydroxyethoxy)-2-methylphenylacetone and TiO_2_. The reaction equation is shown in Figure 1.

A small amount of CMC (0.2 g) was transferred multiple times to 20 mL of deionized water and dissolved by ultrasonic oscillation to obtain a transparent and viscous liquid. Then, 0.01 g of nano-TiO_2_ was added and thoroughly mixed to obtain solution A. We then took 0.2 g of 2-hydroxy-4′-(2-hydroxyethoxy)-2-methylphenylacetone, added it to 10 mL of water, and dissolved it by ultrasonic oscillation to obtain a colorless and transparent solution. Then, we added 0.1 g of MBA and 4 g of SPMA, and we dissolved them by ultrasonic oscillation, obtaining solution B. Solutions A and B were mixed evenly and reacted under artificial ultraviolet light for about 4 h until a milky white viscous solid was obtained. The solid was then freeze-dried at −58 °C for about 10 h to obtain a white dry solid.

### 2.3. Characterization

The chemical composition of the samples was analyzed by infrared spectroscopy using an IR Prestige-21 Fourier-Transform Infrared Spectrometer (FTIR, Shimadzu Corporation, Kyoto, Japan), with a wavenumber range of 400–4000 cm^−1^ and 16 scans with a resolution of 4 cm^−1^ for the test conditions. The surface morphology of the samples after gold spraying to enhance their conductivity was studied using a scanning electron microscope (SEM, SNE-3000, SEC, Suwon, Republic of Korea). Thermal stability analysis was carried out using a comprehensive thermal performance analyzer (TG, TG/DTA 6300, HITACHI, Tokyo, Japan) from 50 °C to 550 °C at a temperature rise rate of 10 °C/min.

### 2.4. Adsorption Performance Testing of Copolymers

Absorbance testing was analyzed by an UV–visible spectrophotometer (UV2600, Shimadzu Corporation, Kyoto, Japan). To explore the optimal adsorption conditions for the ternary copolymer P(CMC-SPMA-MBA), a dye solution was prepared, and adsorption experiments were conducted to investigate the effects of dye type, adsorption time, initial dye concentration, copolymer dosage, adsorption temperature, and acid–base environment on the adsorption performance.

#### 2.4.1. The Influence of Dye Types on Adsorption Performance

We prepared a soap yellow dye solution and a Rhodamine B dye solution with a pH of 3 and 10, respectively, and a concentration of 15 mg/L. We added 5 mg/10 mL of copolymer to each solution and reacted them at room temperature for 20 min. Then, we took the supernatant obtained after centrifugation and analyzed it using a UV–visible spectrophotometer to investigate the effect of dye type on the adsorption performance of the copolymer through its maximum absorption peak.

#### 2.4.2. The Influence of Adsorption Time on Adsorption Performance

We took 5 mg/10 mL of copolymer in a Rhodamine B dye solution with a concentration of 5 mg/L and a pH of 3. They were reacted at room temperature for 10 min, 20 min, 30 min, 40 min, 50 min, 1 h, 2 h, 4 h, 6 h, and 8 h. Then, we took the supernatant obtained after centrifugation and analyzed it using a UV–visible spectrophotometer to explore the effect of time on the adsorption performance of the copolymer through its maximum absorption peak.

#### 2.4.3. The Influence of Initial Concentration of Dye Solution on Adsorption Performance

We prepared Rhodamine B dye solutions with concentrations of 0.5 mg/L, 1 mg/L, 5 mg/L, 10 mg/L, 15 mg/L, and 20 mg/L, and we controlled the pH of the dye solutions at 3. Then, we added 5 mg/10 mL of copolymer to each of the Rhodamine B dye solutions and reacted them at room temperature for 20 min. The resulting solution was taken from the supernatant obtained after centrifugation and analyzed using a UV–visible spectrophotometer to explore the effect of the initial concentration of the dye solution on the adsorption performance of the copolymer through its maximum absorption peak.

#### 2.4.4. The Influence of Polymer Dosage on Adsorption Performance

We took a Rhodamine B dye solution with a pH of 3 and a concentration of 15 mg/L. Then, we added 5 mg/10 mL, 10 mg/10 mL, 15 mg/10 mL, 20 mg/10 mL, 25 mg/10 mL, and 30 mg/10 mL of copolymers and reacted them at room temperature for 20 min. The resulting solution was taken from the supernatant obtained by centrifugation and analyzed using a UV–visible spectrophotometer to investigate the effect of polymer dosage on the adsorption performance of the copolymers through their maximum absorption peaks.

#### 2.4.5. The Influence of Environmental Temperature on Adsorption Performance

We took a Rhodamine B dye solution with a pH of 3 and a concentration of 15 mg/L. Then, we added 5 mg/10 mL of copolymer and allowed them to react at 5 °C, 15 °C, 25 °C, 35 °C, and 45 °C for 20 min. We then took the supernatant obtained after centrifugation and analyzed it using a UV–visible spectrophotometer to investigate the effect of ambient temperature on the adsorption performance of the copolymer through its maximum absorption peak.

#### 2.4.6. The Influence of the Acid–Base Environment on Adsorption Performance

We took Rhodamine B dye solutions with pH 3 or pH 8 and a concentration of 15 mg/L. We then added 5 mg/10 mL of copolymer to each solution and allowed them to react at 35 °C for 20 min. We took the supernatant obtained after centrifugation and analyzed it using a UV–visible spectrophotometer to explore the effect of the acid–base environment on the adsorption performance of the copolymer through its maximum absorption peak.

### 2.5. Degradation Performance Testing of Copolymers

To investigate the degradation performance of the ternary copolymer P(CMC-SPMA-MBA), a suitable dye solution was prepared, and an adsorption reaction was carried out under appropriate conditions. After separation, the upper clear liquid was removed, and the adsorbed copolymer was illuminated with a UV lamp, with a power of 125 W and a wavelength range of 315–400 nm. Electronic photos were taken at different times to record its changes and analyze the degradation performance of the copolymer.

## 3. Results and Discussion

### 3.1. Characterizations of Structure

**Figure 2:**
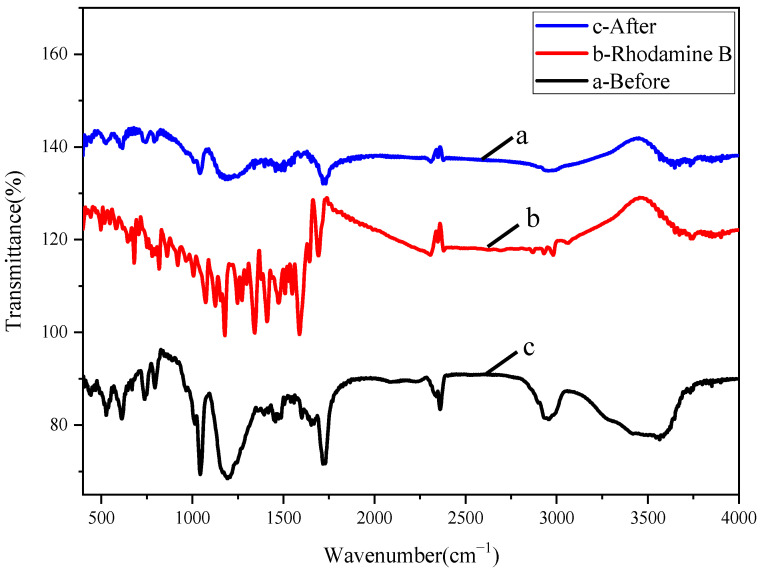
Infrared absorption spectra of P(CMC-SPMA-MBA) before and after dye adsorption: (**a**) Before adsorption. (**b**) Rhodamine B. (**c**) After adsorption.

We used an IR Prestige-21 Fourier-Transform Infrared Spectrometer with a scanning range of 400–4000 cm^−1^. The three peaks at 2984 cm^−1^, 2930 cm^−1^, and 2868 cm^−1^ in Figure 2 belong to the C-H stretching vibration on the benzene ring. The peak at 1694 cm^−1^ belongs to the -C=O stretching vibration, which shifts towards lower frequencies due to its aromatic ketone nature. The peaks at 1587 cm^−1^, 1473 cm^−1^, and 1409 cm^−1^ belong to the characteristic absorption of C=C on the aromatic ring skeleton. There is a possible C-O stretching vibration peak on carboxylic acid at 1342 cm^−1^. The peak at 1510 cm^−1^ is speculated to be caused by C-N. There are two possible peaks at 1178 cm^−1^ due to O-H in-plane deformation and C-O-C anti-stretching vibration.

From Figure 2a,c, it can be seen that the peak at 3563 cm^−1^ is not sharp, which may be caused by the O-H stretching vibration of water molecules on the surface of P(CMC-SPMA-MBA). There are two possible peaks at 2957 cm^−1^: the antisymmetric stretching vibration peak of -CH_3_, and the stretching vibration peak of =CH. The absorption peak at 1728 cm^−1^ is caused by the stretching vibration of -C=O. The peaks at 1194 cm^−1^ and 1043 cm^−1^ may be caused by the stretching vibrations of S=O, C-O, and C-O-C. The peaks at 795 cm^−1^ and 739 cm^−1^ belong to the out-of-plane gamma deformation vibrations of CH. The peaks at 613 cm^−1^ and 529 cm^−1^ may be Ti-O-Ti vibrational absorption peaks. After the copolymer adsorbs the dye, the peak intensity of the P(CMC-SPMA-MBA) spectrum generally changes, and there is a small shift in the peak position, with no new characteristic peaks appearing. The adsorption of Rhodamine B by P(CMC-SPMA-MBA) is not simply physical or chemical adsorption.

### 3.2. Thermal Properties

**Figure 3:**
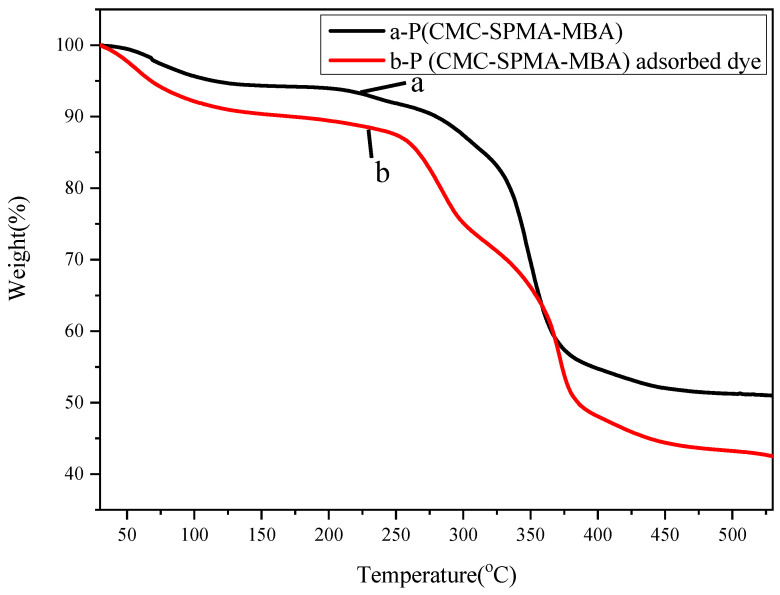
Thermal analysis chart of P(CMC-SPMA-MBA): (**a)** Before adsorption and (**b**) after adsorption.

Thermal properties were studied by a TG analyzer, as can be seen from Figure 3. In Figure 3a, the thermal degradation of P(CMC-SPMA-MBA) before adsorption is mainly divided into two stages. When the temperature rises from 60 °C to 168 °C, there is a slight decrease in weight, with a loss of about 6.06%, which may be caused by the evaporation of water in the network structure. When the temperature rises from 253 °C to 505 °C, the weight decreases significantly, and the fastest decrease occurs at 351.59 °C, with a rate of 1003.40 μg/min. At this stage, the mass loss is about 42.67%, which may be due to the breakage of C-O-C glycosidic chains in carboxymethyl cellulose sodium, resulting in a residual mass of 53.93% of the initial value. Its thermal decomposition temperature is around 310 °C, indicating that polymers below 300 °C can still maintain good thermal stability. In Figure 3b, the thermal degradation of P(CMC-SPMA-MBA) after dye adsorption is mainly divided into two stages. From 30 °C to 167 °C, there is a small amount of weight loss in the material, and the fastest loss occurs at 60.15 °C, with a rate of up to 142.16 μg/min. In the first stage, the evaporation of water in the network structure reduces the mass by 9.96%. When the temperature rises to 318 °C, the weight decreases by 17.46% again. At this stage, when the temperature reaches 287.4 °C, the fastest weight loss occurs at a rate of 375.46 μg/min. When the temperature reaches around 493 °C, the product quality significantly decreases, and the loss rate increases rapidly with increasing temperature. When the temperature is 372.9 °C, the loss rate reaches its maximum of 865.06 μg/min at this stage. Then, as the temperature increases, the trend gradually becomes flat, possibly due to the slow oxidation of P(CMC-SPMA-MBA) after carbonization, and the final residual amount is 41.75% of the initial amount. The thermal decomposition temperature of the copolymer is about 260 °C, and the product can still maintain good thermal stability, indicating that the thermal stability of the copolymer has decreased after adsorption but still remains at a relatively high level.

### 3.3. Characterizations of Surface Morphology

**Figure 4:**
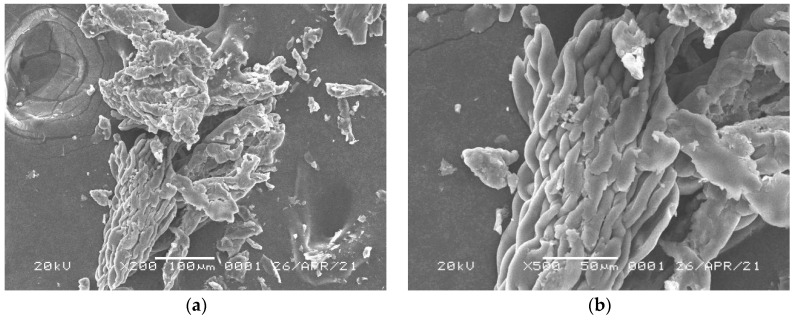

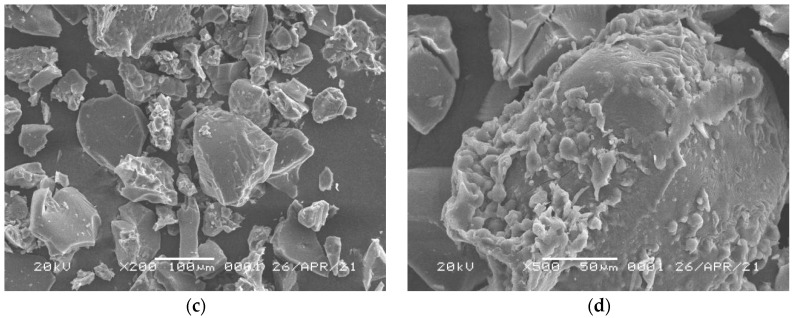
SEM images of P(CMC-SPMA-MBA): (**a**) before adsorption (200×); (**b**) before adsorption (500 times); (**c**) after adsorption (200 times); (**d**) after adsorption (500 times).

Figure 4a,b are SEM images of P(CMC-SPMA-MBA) at 200× and 500× magnification, respectively. From the image, it can be seen that the surface of the copolymer is smooth and rough, with a bundle-like structure and distributed pores. The increase in surface area is beneficial for improving the adsorption performance of the copolymer, and TiO_2_ aggregates are formed on the surface. Figure 4c,d show the images of the solid obtained by adsorbing 5 mg/10 mL of P(CMC-SPMA-MBA) in 15 mg/L Rhodamine B dye solution at 35 °C and pH = 3 for 20 min, and then separating and drying at 200× and 500× magnification, respectively. From the image, it can be seen that the surface of the material is relatively smooth and slightly rough, forming larger aggregates that adsorb Rhodamine B. This also proves that the copolymer does indeed have an adsorption effect on Rhodamine B.

### 3.4. Adsorption Performance of Copolymers

#### 3.4.1. The Influence of Dye Types on Adsorption Performance

To investigate the effects of dye types on adsorption performance, Rhodamine B was selected as the cationic dye and soap yellow as the anionic dye in the experiment. Soap yellow dye solution and Rhodamine B dye solution were prepared at pH 3 and pH 10, respectively, at a concentration of 15 mg/L. Then, 10 mL of each solution was added to 5 mg of copolymer and reacted at room temperature for 20 min. The effect of dye types on the adsorption performance of the copolymer was analyzed by UV–visible light in the range of 380–780 nm.

**Figure 5:**
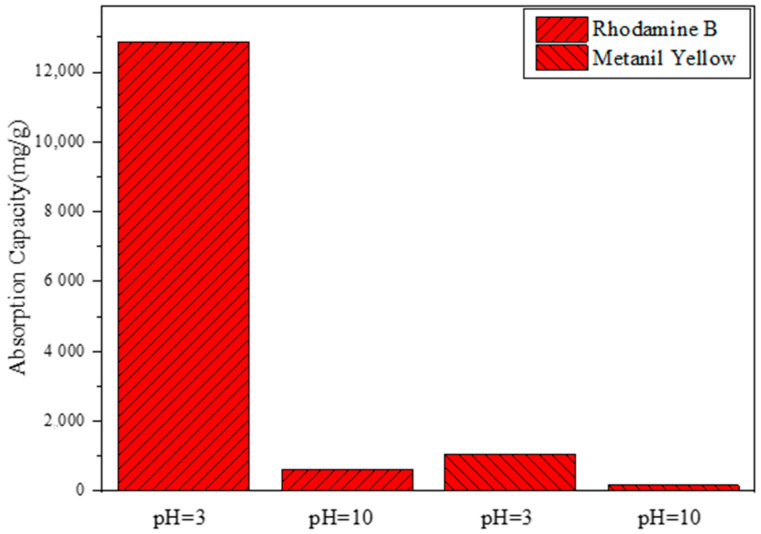
The influence of different dyes on adsorption performance under different acid–base environments.

Through the comparative analysis of Figure 5, it can be seen that, in acidic environments, the copolymer has a poor adsorption effect on soap yellow, with an adsorption capacity of 633.53 mg/g, while it has a relatively significant adsorption effect on Rhodamine B, with an adsorption capacity of up to 12,869.02 mg/g. Through the comparative analysis of Figure 5, it was found that, under alkaline conditions, the copolymer had almost no adsorption effect on soap yellow, with an adsorption capacity of only 175.61 mg/g. In contrast, the adsorption effect for Rhodamine B was good, with an adsorption capacity of up to 1053.69 mg/g. As Rhodamine B is a cationic dye, it exists in a cationic form in acidic environments, and the P(CMC-SPMA-MBA) contains sulfonic acid groups, which attract the cations in Rhodamine B, resulting in the highest adsorption capacity.

#### 3.4.2. The Influence of Reaction Time on Adsorption Performance

Fast dye absorption and high adsorption capacity are the most ideal properties of adsorbents. To investigate the effect of reaction time on adsorption performance, 10 mL of copolymer was taken and added to 10 mg of 5 mg/L Rhodamine B dye solution. The reaction was carried out at room temperature for 10 min, 20 min, 30 min, 40 min, 50 min, 1 h, 2 h, 4 h, 6 h, and 8 h. The effect of reaction time on the adsorption performance of the copolymer was analyzed by UV–visible light in the wavelength range of 380 nm–780 nm. According to the following equation [27], the dye adsorption amount *q_e_* (mg/g) per unit mass of copolymer can be calculated:

(1) $$q_{e}=\frac{(C_{0}-C_{e})V}{W}$$

where, *C*_0_ is the initial concentration of the dye solution (mg/L), *V* is the volume of the dye solution used (L), *C_e_* is the calculated concentration of the adsorbed dye solution (mg/L), and *W* is the mass of the copolymer used for adsorption (g).

**Figure 6:**
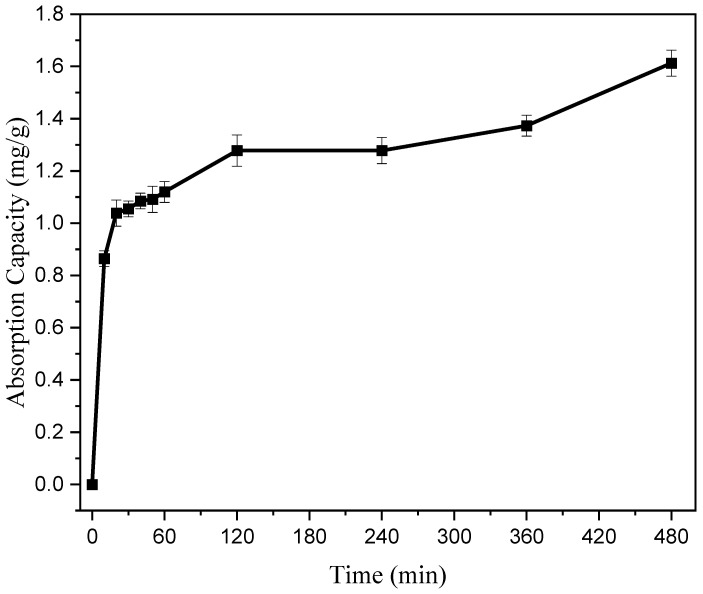
Effect of reaction time on adsorption performance.

According to Figure 6, under the condition of keeping other factors constant, as the reaction time gradually increases, the adsorption rate of the copolymer is higher within 60 min before adsorption, and its adsorption capacity rapidly increases. This may be attributed to the existence of a large number of vacant adsorption sites available for initial adsorption. When the adsorption reaction time exceeds 20 min, the increase in adsorption capacity slows down, and the increase in adsorption rate slows down, indicating that a certain mass of copolymer needs at least 8 h to reach adsorption saturation. When the reaction time is 8 h and reaches saturation, the maximum adsorption capacity is 1.61 mg/g.

#### 3.4.3. The Influence of the Initial Concentration of Dye Solution on Adsorption Performance

To investigate the effect of the initial concentration of dye solution (Rhodamine B) on adsorption performance, 10 mg/10 mL of copolymer was added to a 5 mg/L Rhodamine B dye solution and reacted at room temperature for 20 min. The effect of the initial concentration of dye solution (Rhodamine B) on the adsorption performance of the copolymer was analyzed by UV–visible light in the range of 380–780 nm.

**Figure 7:**
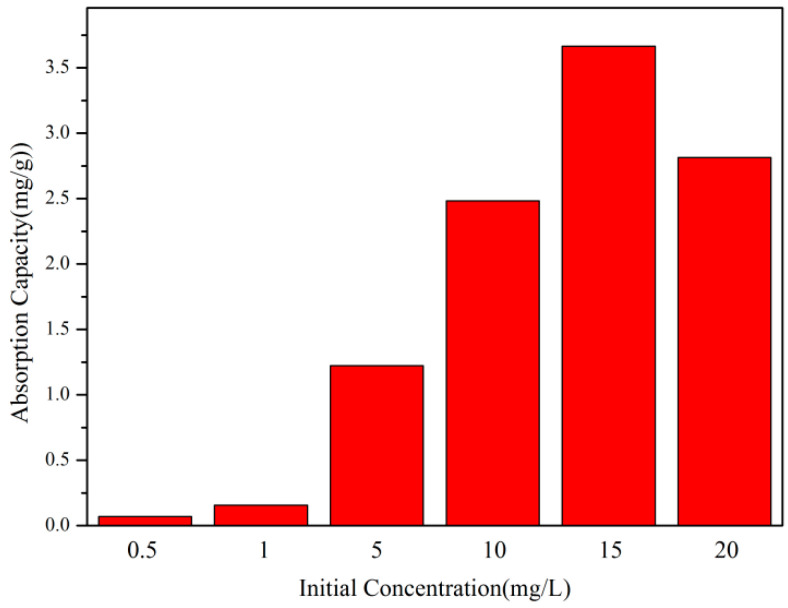
The influence of the initial concentration of dye solution on adsorption performance.

According to Figure 7, under the condition of keeping other factors constant, as the initial concentration of the dye solution increases, the adsorption capacity of the copolymer per unit mass continues to increase. When the initial concentration of the dye solution exceeds 15 mg/L, the adsorption capacity decreases. When the concentration is 15 mg/L, the adsorption capacity is the highest (3.67 mg/g). The initial concentration of the dye solution gradually increases, and the adsorption capacity increases linearly. When the initial concentration of the dye solution exceeds 20 mg/L, the increase in adsorption capacity accelerates, and when it exceeds 20 mg/L, the adsorption capacity decreases. The reason for this is that when the dye solution concentration is low, there are still some adsorption sites, while when the dye concentration is high, the driving force for mass transfer is higher.

#### 3.4.4. The Influence of Polymer Dosage on Adsorption Performance

In order to avoid excessive use of adsorbents, it is necessary to calculate the optimal dosage of adsorbents to effectively remove dyes. To investigate the effect of polymer dosage on adsorption performance, copolymers of 5 mg/10 mL, 10 mg/10 mL, 15 mg/10 mL, 20 mg/10 mL, 25 mg/10 mL, and 30 mg/10 mL were taken and added to a 5 mg/L Rhodamine B dye solution. The reaction was carried out at room temperature for 20 min, and the effect of the copolymer dosage on the copolymer adsorption performance was analyzed by UV–visible spectrophotometry.

**Figure 8:**
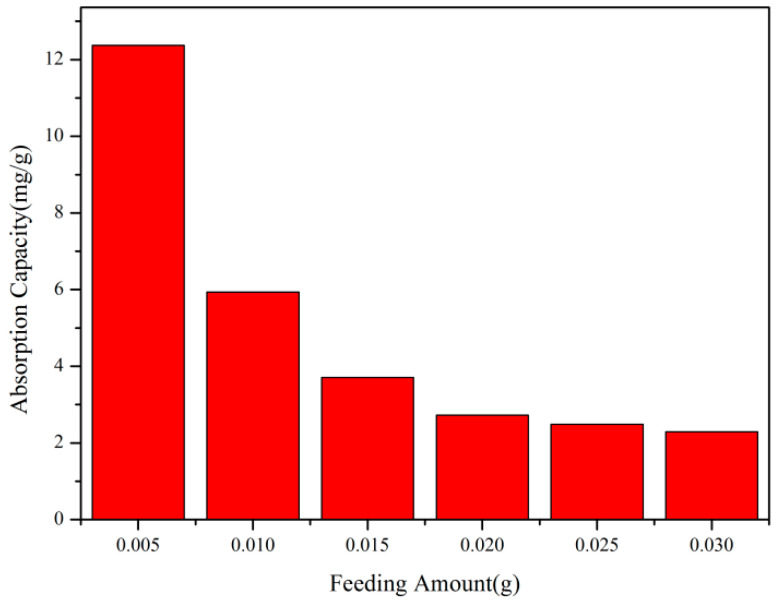
The influence of polymer dosage on adsorption performance.

According to Figure 8, it can be seen that, keeping other factors constant, the adsorption reaction was carried out in 10 mL of Rhodamine B dye solution. When the dosage was within the range of 0.005–0.01 g, the adsorption capacity per unit mass of copolymer increased rapidly with the increase in the copolymer dosage. When the dosage was within the range of 0.01–0.03 g, the adsorption capacity per unit mass of copolymer increased rapidly with the increase in the copolymer dosage. When the dosage was 0.005 g, the adsorption capacity reached a maximum of 12.37 mg/g.

#### 3.4.5. The Influence of Environmental Temperature on Adsorption Performance

To investigate the effect of environmental temperature on adsorption performance, 5 mg of copolymer was taken and added to 10 mL of 5 mg/L Rhodamine B dye solution. The reaction time was controlled at 20 min, and the experiments were conducted at reaction temperatures of 5 °C, 15 °C, 25 °C, 35 °C, and 45 °C. We analyzed the effect of reaction temperature on the adsorption performance of the copolymers in the wavelength range of 380 nm–780 nm, using a UV–visible spectrophotometer.

**Figure 9:**
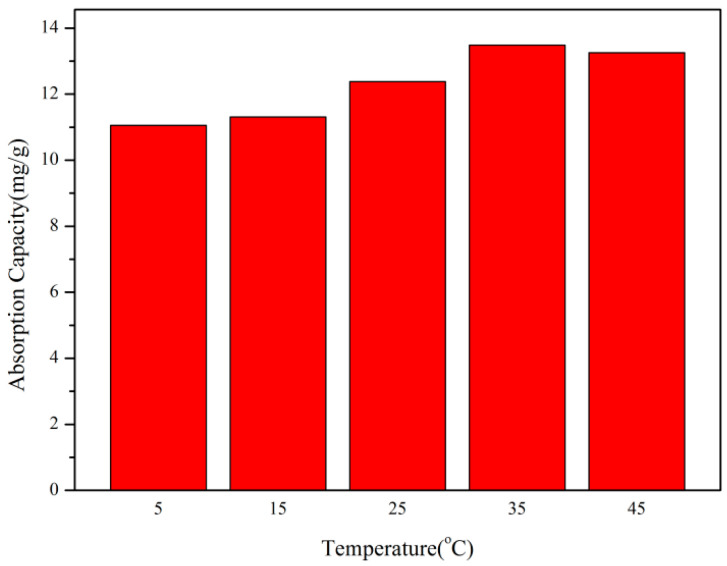
Effect of temperature on adsorption performance.

According to Figure 9, it can be seen that, under the condition of keeping other factors constant, when the reaction temperature is in the range of 5–35 °C, the adsorption amount of copolymer per unit mass gradually increases with the increase in the reaction temperature. When the reaction temperature is within the range of 35–45 °C, the adsorption capacity of the copolymer per unit mass decreases with the increase in the reaction temperature. When the temperature of the reaction environment is 35 °C, the highest adsorption capacity is 13.48 mg/g. This indicates that high temperatures are beneficial for the adsorption of cationic dyes on copolymers. The positive correlation between environmental temperature and dye adsorption can be attributed to the increased permeability of dye molecules on copolymers. With the increase in temperature, the solvent molecules remaining in the gel will be removed, and the dye molecules will obtain more adsorption sites, thus increasing the adsorption capacity of the dye [28], and the diffusion of the adsorbent molecules from the dye solution to the adsorbent will increase faster.

#### 3.4.6. The Influence of the Acid–Base Environment on Adsorption Performance

The pH is an important parameter that controls the surface charge of adsorbents and the degree of dye ionization. To investigate the effect of the acid–base environment on adsorption performance, 5 mg of copolymer was taken and added to 10 mL of 5 mg/L Rhodamine B dye solution prepared at pH 3 and pH 10. The adsorption reaction was carried out at 35 °C for 20 min, and the effect of the acid–base environment on the adsorption performance of the copolymer was analyzed by UV–visible spectrophotometry.

**Figure 10:**
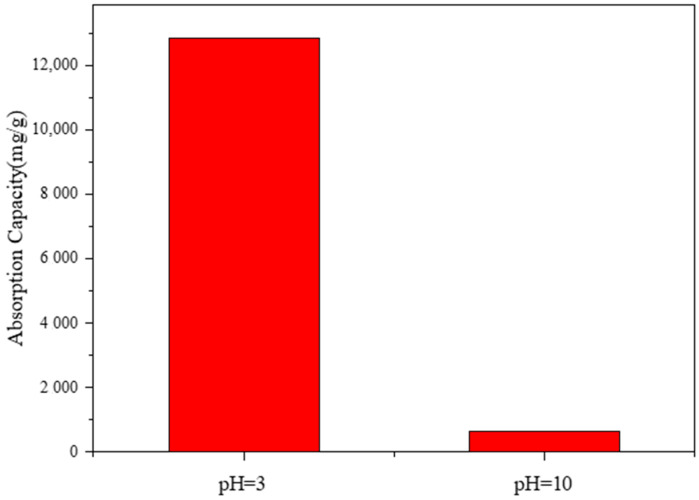
The influence of different acid–base environments on the adsorption performance of Rhodamine B.

According to Figure 10, it can be seen that, under the condition of keeping other factors constant, when the reaction is carried out in an alkaline environment, the adsorption capacity of the copolymer per unit mass is very small. When the reaction is carried out in an acidic environment, the adsorption capacity of the copolymer per unit mass is large, and the phenomenon is more obvious. The pH is an important parameter that controls the surface charge of adsorbents and the degree of dye ionization. In acidic environments, due to the shift in the ionization equilibrium, the surface of copolymers becomes negatively charged, thereby increasing the available sites for negative ions. In the presence of TiO_2_ semiconductor particles, Rhodamine B dye molecules are excited by a light source to become positively charged free radicals, and an acidic environment is more conducive to the generation of free radicals, which enhances the interaction between these free radicals and negatively charged copolymers. Therefore, negatively charged ion functional groups are advantageous for ion interactions with cationic dye molecules.

### 3.5. Degradation Performance of Copolymers

**Figure 11:**
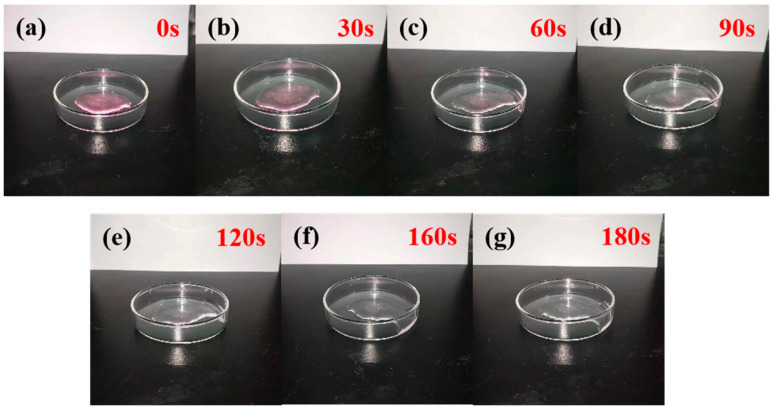
Photo of the process of changes in the adsorption of Rhodamine B by P(CMC-SPMA-MBA) under UV irradiation: (**a**) 0 s; (**b**) 30 s; (**c**) 60 s; (**d**) 90 s; (**e**) 120 s; (**f**) 160 s; (**g**) 180 s.

We took 0.02 g of P(CMC-SPMA-MBA) and placed it in 10 mL of 5 mg/L Rhodamine B staining solution, controlling its pH at 3, and then we shook the solution thoroughly at 35 °C for 20 min. After centrifugation, we removed the upper clear liquid and irradiated it with UV light for 30 s, 60 s, 90 s, 120 s, 150 s, or 180 s. The change process is shown in Figure 11. After 180 s of irradiation, P(CMC-SPMA-MBA) adsorbed with Rhodamine B was completely degraded and turned into a colorless and transparent liquid. The results showed that TiO_2_ had a great influence on the photocatalytic degradation of the Rhodamine B and copolymers.

### 3.6. Comparison of Various Adsorbents

A comparison of the maximum adsorption capacities between prepared P(CMC-SPMA-MBA) and previously reported adsorbents is summarized in Table 1. As can be seen, the adsorption capacity (*q_e_*) of P(CMC-SPMA-MBA) was apparently better than that of most of the other adsorbents. The high adsorption capacity obtained in this work could be mainly attributed to the large surface area, leading to large adsorption sites, and the special structure, resulting in good adsorption properties for Rhodamine B removal.

## 4. Conclusions

This work used molecular structure design and aqueous solution polymerization methods, with cellulose as the skeleton, to react CMC, MBA, and SPMA monomers under the initiation of a photoinitiator to obtain P(CMC-SPMA-MBA) copolymer. We explored the effects of dye types, environmental temperature, acidity and alkalinity of the adsorption environment, polymer dosage, initial dye concentration, and reaction time on the adsorption performance of copolymers. The results showed that P(CMC-SPMA-MBA) had a much better adsorption effect on Rhodamine B (cationic dye) than on soap yellow (anionic dye). The longer the adsorption reaction takes place, the greater the adsorption capacity, and within the first 60 min, the adsorption rate is higher. As the initial concentration of the dye solution increases, the adsorption capacity first increases and then decreases, reaching its maximum at 15 mg/L. As the dosage of P(CMC-SPMA-MBA) continues to increase, the dosage decreases and reaches its maximum at 5 mg/10 mL. As the ambient temperature increases, the adsorption capacity first increases and then decreases, reaching its maximum at 35 °C. P(CMC-SPMA-MBA) has an adsorption effect on Rhodamine B. Although it is effective in both acidic and alkaline environments, its adsorption capacity in acidic environments is much higher than in alkaline environments. The polymer adsorbed with the dye was degraded by UV irradiation, avoiding secondary pollution caused by recycling.

## Figures and Tables

**Table 1:** Maximum adsorption capacities of different adsorbents.

Adsorbent	Adsorption Capacity/mg/g	Ref.
PEGDMA	6	[29]
Kaoli	10.4	[30]
3D rGO-hydrogel	7.85	[31]
Natural Akadama clay	4.29	[32]
P(CMC-SPMA-MBA)	13.48	This work

## Data Availability

All data included in this work are available upon request by contact with the corresponding author.
